# Insights into Cold Plasma Treatment on the Cereal and Legume Proteins Modification: Principle, Mechanism, and Application

**DOI:** 10.3390/foods13101522

**Published:** 2024-05-14

**Authors:** Bin Li, Lianxin Peng, Yanan Cao, Siyao Liu, Yuchen Zhu, Jianguo Dou, Zhen Yang, Chenguang Zhou

**Affiliations:** 1 School of Food and Biological Engineering, Jiangsu University, Zhenjiang 212013, China; 2 Key Laboratory of Coarse Cereal Processing of Ministry of Agriculture and Rural Affairs, Chengdu University, Chengdu 610106, China; 3 School of Pharmacy, Jiangsu University, Zhenjiang 212013, China; 4 Key Laboratory of Nuclear Agricultural Sciences of Ministry of Agriculture and Zhejiang Province, Institute of Nuclear Agricultural Sciences, Zhejiang University, Hangzhou 310058, China

**Keywords:** cold plasma, proteins, modification, functional properties

## Abstract

Cereal and legume proteins, pivotal for human health, significantly influence the quality and stability of processed foods. Despite their importance, the inherent limited functional properties of these natural proteins constrain their utility across various sectors, including the food, packaging, and pharmaceutical industries. Enhancing functional attributes of cereal and legume proteins through scientific and technological interventions is essential to broadening their application. Cold plasma (CP) technology, characterized by its non-toxic, non-thermal nature, presents numerous benefits such as low operational temperatures, lack of external chemical reagents, and cost-effectiveness. It holds the promise of improving proteins’ functionality while maximally retaining their nutritional content. This review delves into the pros and cons of different cold plasma generation techniques, elucidates the underlying mechanisms of protein modification via CP, and thoroughly examines research on the application of cold plasma in augmenting the functional properties of proteins. The aim is to furnish theoretical foundations for leveraging CP technology in the modification of cereal and legume proteins, thereby enhancing their practical applicability in diverse industries.

## 1. Introduction

Grains and legumes are valuable sources of plant proteins, featuring a diverse array of proteins such as gluten, zein, legumin, β-conglycinin, glycinin, and prolamins, among others. These proteins significantly contribute to the functional characteristics that are essential for maintaining quality during food processing [1]. However, these natural proteins may exhibit limitations, such as poor solubility and allergenicity, which restrict their use in the food industry [2]. To overcome these limitations, food processing commonly employs enzymes and chemical and physical methods to enhance the functional characteristics of proteins. These include solubility, gelation, emulsification, and foaming, which are critical to meeting production requirements [3]. Consequently, there is a growing interest in employing eco-friendly non-thermal methods to modify protein structures and improve their functional characteristics, which has become a research hotspot in food science.

Numerous non-thermal techniques have been developed for protein modification, including high pressure [4], ultrasound [5], pulsed electric fields [6], irradiation [7], and supercritical fluids [8]. These methods, which preserve nutritional and sensory qualities by avoiding thermal degradation, have their unique benefits and drawbacks. High pressure, ultrasound, and pulsed electric fields are noted for efficiently altering protein conformation while maintaining food quality. However, methods like irradiation and supercritical fluids often require high energy inputs and specialized equipment, making them costly and less accessible. Additionally, certain techniques might induce undesirable changes in protein structures, affecting functional properties, and face regulatory and consumer acceptance challenges. In contrast, cold plasma technology is gaining attention for its low-temperature operation, energy efficiency, and ability to preserve heat-sensitive compounds [9,10]. 

**Figure 1:**
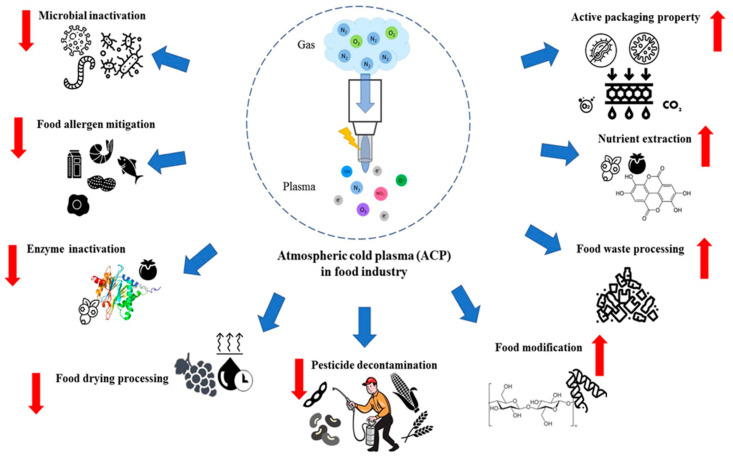
Cold plasma (CP) treatment advancements in food processing. Reprinted from ref. [22].

Cold plasma (CP) technology, originally utilized in the electronics industry and for improving polymer material surfaces [11,12,13], has expanded its applications to the food industry. The versatility of cold plasma is evident in its diverse applications within food technology, where it provides numerous benefits as detailed in Figure 1. Key applications of CP in this sector include the microbial decontamination of food products [14], processing of packaging materials [15], mitigation of food allergens [16], and functionality modification of food materials [17]. Beyond these applications, CP technology is increasingly utilized in the food industry for the degradation of mycotoxins [18], enzyme inactivation [19], augmentation of bio-active compound concentrations [20], and pesticide decontamination [21].

As an innovative non-thermal food processing technology, cold plasma (CP) offers new possibilities for sterilizing volatile and heat-sensitive foods and demonstrates significant potential in enhancing protein functional characteristics [19,23]. Cold plasma modifies protein structures through the production of reactive oxygen species (ROS), reactive nitrogen species (RNS), free radicals, and high-speed electrons. These reactive species induce changes in proteins, such as chemical bond formation, amino acid side chain oxidation, and polypeptide chain cross-linking, thereby improving protein functionality [24,25]. Therefore, investigating the changes in protein functional characteristics following cold plasma treatment is essential for unlocking the practical value of this technology. 

This review aims to provide an overview of the application of cold plasma technology in modifying grain and legume proteins. It briefly discusses the mechanism of CP-modified proteins and the relationship between protein structure and function, offering valuable references for future research in this field. The primary goal of this review is to elucidate the modifications in functional characteristics of proteins in grains and legumes following treatment with cold plasma technology, thereby enhancing their practical utility in the food industry. This examination involves a detailed analysis of the structural modifications induced by cold plasma, exploring the consequential changes in their functional properties. Furthermore, the underlying mechanisms by which cold plasma modifies proteins and the implications of these changes on the relationship between protein structure and function are discussed. This review not only synthesizes current findings but also provides crucial references that pave the way for future research in this rapidly evolving field.

To comprehensively understand the developments in CP technology and its application in enhancing the functionality of cereal and legume proteins, a literature search was conducted across major databases, including PubMed, Scopus, and Web of Science. The search utilized the keywords “cold plasma technology”, “protein functionality enhancement”, “cereal proteins”, and “legume proteins”. These terms were combined with Boolean operators to refine the search scope, focusing on articles that discuss technological interventions in protein modification. The inclusion criteria limited the search to peer-reviewed articles published in English from 2010 to the present, capturing the most relevant and recent advancements. 

## 2. Fundamentals of Cold Plasma

Plasma, the fourth state of matter, is formed by ionizing a neutral gas under high pressure. This process creates an ionized gas comprising molecules in both excited and ground states, atoms, free electrons, and ions carrying positive and negative charges [26]. Despite the charged components, plasma remains electrically neutral due to the balance of positive and negative charges. Distinct from solids, liquids, and gases, plasma’s generation requires energy to initiate and sustain the ionization process, which leads to a series of excitation, de-excitation, and further ionization reactions [13].

**Figure 2:**
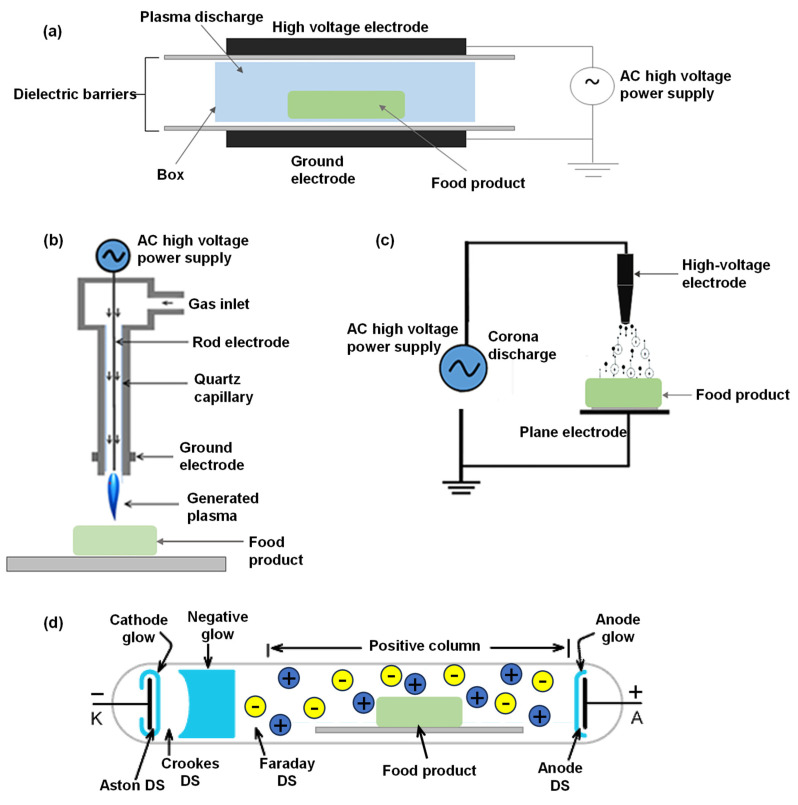
Different configurations of cold plasma designs: (**a**) dielectric barrier discharge, (**b**) plasma jet, (**c**) corona discharge, and (**d**) glow discharge. Reprinted from refs. [31,32].

The generation of plasma involves energy transfer and is classified based on the thermal equilibrium between ion and electron temperatures and the degree of ionization [27]. Thermal plasma occurs when these temperatures are in equilibrium, resulting in nearly complete ionization of the gas. In contrast, cold plasma exists in a non-equilibrium state, with the electron temperature significantly exceeding the ion temperature, leading to partial ionization [28]. This form of plasma is particularly relevant for low-temperature food processing applications due to its unique advantages. Plasma generation can be achieved through various methods, including electric fields, heating, and high-energy radiation [29]. In laboratory research, ionization typically occurs through collisions involving accelerated electrons, ions, or high-energy neutral particles. Common systems for generating cold plasma include dielectric barrier discharge, corona discharge, sliding arc (glow) discharge, and plasma jet [30]. Detailed configurations of these distinct cold plasma designs are illustrated in Figure 2, providing a visual comparison and technical differentiation.

### 2.1. Dielectric Barrier Discharge (DBD)

In DBD systems, an insulating medium is placed between the discharge electrodes. The application of a high-voltage alternating current across the electrodes capable of breaking down the intermediate discharge gas establishes a conductive channel, and space charges accumulate on the dielectric surface [33]. These charges form an electric field opposing the external field, limiting current growth and preventing sparks and arcs [34]. The resulting micro-discharge is discontinuous and semi-cyclical, with the dielectric acting as a barrier during the other half of the cycle. This process leads to the breakdown of the working gas and the production of numerous free radicals and active particles, forming low-temperature plasma [35]. The dielectric in DBD systems can be a single layer, placed between the electrodes or wrapped around one electrode, or a double layer, typically used to cover both electrodes and isolate them from the sample. This arrangement prevents electrode wear and sample contamination. Common materials for the insulating medium include quartz, glass, ceramics, thin enamel and polymers. In cases with specific requirements, additional protective layers or functional coatings may be applied [33]. The high voltage frequency applied across the electrodes typically ranges from several hundred to tens of thousands of hertz [36]. DBD’s ability to generate plasma at atmospheric pressure, coupled with its simple structure, small size, portability, low manufacturing cost, and low power consumption, has led to its widespread use in food research, making it the most popular plasma generation method in this field [35]. DBD is a widely utilized method for generating CP specifically for protein modification. Recent research has highlighted the efficacy of CP treatment in enhancing the physicochemical and functional properties of proteins, particularly in reducing their allergenicity. Significant findings include the reduction of allergenic properties in various proteins such as peanut protein isolate [37], shrimp protein [38], and milk protein [39]. Meinlschmidt et al. [37] demonstrated that plasma treatment at 11 kV for 10 min reduced the immunoreactivity of soy protein isolate (SPI) by 91–100%. In a related study, Zhang et al. [40] observed that DBD plasma treatment at 40 kV for 10 min led to a reduction of up to 75% in the IgE-binding capability of SPI.

### 2.2. Atmospheric Pressure Plasma Jet (APPJ)

The APPJ is an innovative technology characterized by a unique electrode arrangement, as depicted in Figure 2. In this setup, gas flows between two electrodes, becomes ionized in the process, and is subsequently expelled from the ionization chamber [41]. A distinct feature of APPJ is its operation in open space, which eliminates size constraints on the objects being treated. This flexibility allows the plasma jet to extend over several centimeters, reaching lengths greater than 10 cm, and to produce plasma enriched with a diverse mix of chemicals, charged particles, free radicals, as well as emitting heat and ultraviolet radiation [42]. Unlike low-pressure plasma systems, APPJ obviates the need for elaborate vacuum setups, thereby offering a cost-effective solution. The design of APPJ ensures that plasma, propelled by a vigorous airflow, is directly discharged into the atmosphere, distancing the high-voltage electrode from the plasma to enhance operational safety [29]. Its versatility in design allows for adaptation to varied operational conditions and is not constrained by spatial limitations, enabling the treatment of diverse materials ranging from polymers to metals without shape restrictions [41]. Under specific non-equilibrium conditions, the plasma’s gas temperature can closely approximate room temperature, further broadening APPJ’s applicability in areas such as material surface treatment, film deposition, and biomedical applications, underscoring its significant advantages and utility in multiple domains [42]. The APPJ produces a stable and uniform discharge suitable for applications at atmospheric pressure [43]. While this plasma is effective, its coverage is limited to smaller areas. For larger surface treatments, an arrangement of multiple jets is typically necessary [44]. Lee et al. [45] developed a pilot-scale APPJ system for the inactivation of natural bacteria in particulate foods (rice germ, black pepper powder, and sesame). This system integrates a pneumatic suction conveyor capable of handling up to 226 kg/h. Specifically, the treatment compartment is equipped with four plasma jets, each operating at an output voltage of 1 kV, a frequency of 30 Hz, and a plasma discharge length of 18 mm from the nozzle. Further research involving a plasma jet reactor demonstrated the utility of cold plasma (CP) in food preservation. The PJ reactor operates with 12 kHz pulsed DC and a 15-kV power supply. This configuration was applied to walnuts to study the effects of CP on the stability of total phenolic content and antioxidant activity [46].

### 2.3. Corona Discharge

Corona discharge typically occurs on electrodes with small curvature radii, such as needle-shaped or filament electrodes. At or above atmospheric pressure, a strong electric field forms near the electrode, ionizing the surrounding air and initiating the corona discharge reaction. Electrons near the electrode gain significant energy, ionizing gas molecules through collisions and producing more electrons, resulting in the electron avalanche effect [47]. This phenomenon is a localized, self-sustained discharge in a gas medium within an inhomogeneous electric field, often visible as a blue-purple corona around conductors and the edges of strong electric fields [48]. Under high electric fields, the gas undergoes extensive local ionization, while under weak electric fields, it remains non-ionized without breakdown between electrodes, a process known as corona discharge [47]. The properties of the generated plasma are influenced by the shape of the electrode, the applied voltage, and the type of background gas used for ionization. The voltage and the inhomogeneous electric field produced by the electrodes significantly impact the energy and density of electrons, with the electrode effect being more pronounced. The inhomogeneity of the electric field confines the main ionization process to areas near the electrode with high local electric fields, termed the corona region [49]. In the outer area, where the electric field is weaker, ionization is rare or absent, and the current is primarily conducted by the migration of positive and negative ions or electrons, referred to as the migration or peripheral area [50]. Corona discharge, with its low cost and ease of operation, has been applied in microbial purification, surface treatment, and spore inactivation [49]. This technology has demonstrated significant potential in enhancing the quality of agricultural products, including onions [51], tomatoes [52] and kumquat fruits [53]. Additionally, both DC and pulsed corona discharges have shown effectiveness against Streptococci biofilms and B. cereus spores on plastic surfaces [54]. In a detailed study, researchers [55] reported substantial bacterial population reductions—achieving 2.4 logs in 10 min with DC discharges and 3.3 logs in just 2 min with pulsed corona discharges. Further investigations have revealed that corona discharge plasma not only alters bacterial cell morphology but also causes significant damage to the internal structure of spores, particularly in Penicillium expansum aerosols [56]. These findings underscore the effectiveness of corona discharge plasma in microbial control and its impact on microorganism structure at the cellular level.

### 2.4. Glow Discharge

Glow discharge, also known as high-frequency discharge, occurs in low-pressure gas and is characterized by the emission of a glowing light during discharge [41]. This phenomenon is notable for its ability to maintain a stable voltage across a wide range of discharge currents [41]. In the process of glow discharge, electrons in the gas are accelerated by the electric field when the voltage between the electrodes is high. These electrons gain sufficient energy to excite and ionize gas molecules, leading to the creation of an electron avalanche [29]. Distinctively, glow discharge operates at low current intensities, typically only a few milliamps, and maintains a low temperature of the discharge gas. This type of plasma is more homogeneous compared to other discharge plasmas and is capable of generating plasma over large volumes at low temperatures [57]. One of the principal advantages of glow discharge plasma is its ability to generate a continuous stream of gas plasma, which is then introduced into a spacious chamber for sample treatment. This method facilitates the processing of large sample volumes. However, a significant limitation of this technique is the requirement for maintaining low pressures (<0.5 bar) within the treatment chamber [58].

A typical direct-current glow discharge consists of four main regions, as depicted in Figure 2d: the cathode region, the transition region, the positive column region, and the anode region. The cathode region is comprised of the Aston dark space, the cathode glow, and the cathode dark space, all of which exhibit weak luminescence and short lengths [42]. The transition region contains the negative glow and the Faraday dark space. The negative glow, also known as the plasma region, is the brightest part of the discharge space due to the roughly equal amounts of positive and negative charges [41]. Electrons that are accelerated in the cathode region gain enough energy to cause ionization and excitation in the negative glow, resulting in strong luminescence. The Faraday dark space, characterized by a lower ion concentration and weaker recombination, appears as a darker area due to the reduced energy of electrons. The positive column region, with equal concentrations of electrons and ions, serves to conduct current in the discharge. Its length is determined by the distance between the electrodes [29]. 

While DBD and APPJ have been extensively studied, glow discharge plasma remains less explored in the context of food processing. However, recent investigations have begun to reveal its potential. For instance, Fernandes et al. [59] demonstrated that treating acerola juice with glow discharge plasma using N_2_ at a flow rate of 10 mL/min for 10 min significantly increased the content of vitamin A and carotenoids—from 760 IU/L and 2000–3000 μg/L to approximately 3000 IU/L and 7000–8000 μg/L, respectively. Although the underlying mechanisms remain unclear, it is suggested that ionized nitrogen species may disrupt the bond between vitamin A and lipoproteins, thereby increasing the availability of free vitamin A. Further research by Pathan et al. [60] applied glow discharge plasma to enhance protein digestibility in six chickpea cultivars, with treatments administered at 60, 50, and 40 W for durations of 20, 15, and 10 min, respectively. The most significant improvement in digestibility was observed in samples treated for 20 min at 60 W. In another application, orange juice was subjected to glow discharge plasma at flow rates ranging from 10 to 30 mL/min for durations of 10 to 30 min [61]. This treatment facilitated chemical reactions that beneficially altered the juice’s volatile profile, notably increasing the concentration of terpenoid and aldehyde compounds that enhance the fresh-like characteristics of the juice, while reducing off-flavor compounds by 61%.

## 3. Operating Principles of CP

### 3.1. High-Speed Particle Etching Effect

**Figure 3:**
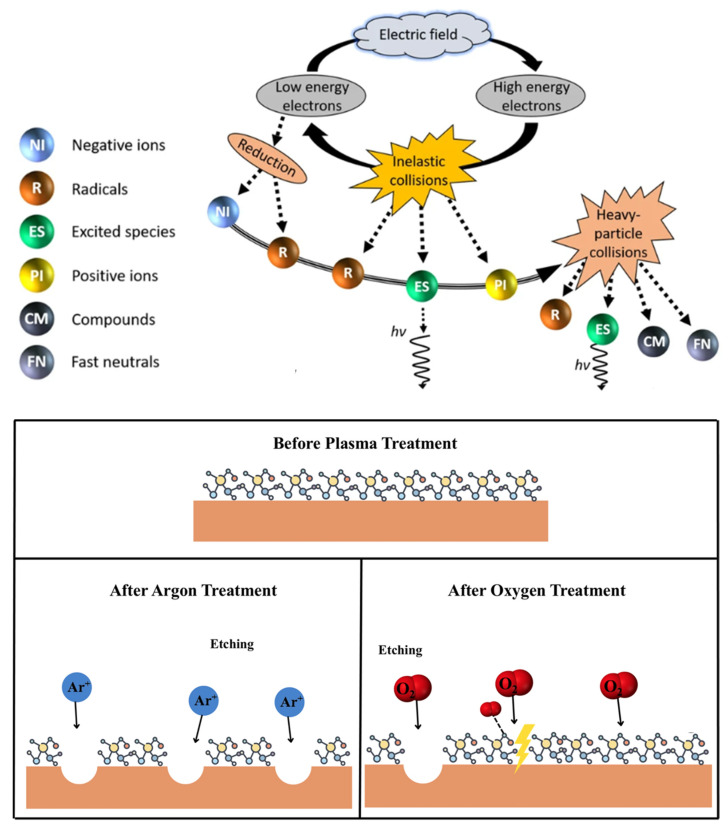
The generation of various high-speed particles from cold plasma [68] and schematic representation of the etching effect of plasma treatment [69]. Reprinted from refs. [68,69].

The generation of cold plasma is marked by a dynamic process of energy transfer. This begins with the acceleration of electrons by an external electric field. These electrons then collide with surrounding gas molecules, leading to their excitation and, eventually, ionization. This sequence results in a significant increase in the electron population. As the process unfolds, certain atoms or molecules gain high levels of energy, transforming into high-energy active particles. These particles engage in inelastic collisions with non-excited species, creating a wide array of active free radicals [62,63]. The mechanisms involved in the production of various high-speed particles from cold plasma and the etching effect of plasma treatment are depicted in Figure 3. Upon exposure to the electric field, these energetic particles bombard the surfaces of proteins. This interaction induces an etching effect and facilitates energy transfer to the surface of the material, promoting the breakage and formation of covalent bonds. The resulting chemical reactions lead to a series of physicochemical changes, including bond disruption and chemical degradation, primarily affecting the protein’s surface [64,65]. This modulation of the surface enhances surface roughness in polymers and creates cavities on protein surfaces, playing a crucial role in protein modification [66]. Moreover, the etching effect reduces particle size, thus increasing the contact area with active particles and improving the modification efficacy [67].

### 3.2. Oxidation Effect of Active Particles

In the context of low-temperature plasma, the electric field acts as a catalyst for the formation of various reactive species such as oxygen free radicals (·O), hydroxyl free radicals (·OH), ozone (O_3_), and nitrogen dioxide (NO_2_) [70]. These reactive species primarily target amino acid residues on protein molecules, inducing structural and functional modifications [71]. One study demonstrated that the reduced IgG/IgE binding capacity of glycine, following medium barrier discharge plasma treatment, was associated with the oxidation of peptide bonds and sensitive amino acids like Trp, Tyr, and Phe [72]. This oxidative process disrupts intermolecular hydrogen bonds and other non-covalent interactions within the amino acids, facilitating protein modification. The main agents driving changes in CP-treated proteins are reactive oxygen and nitrogen species, which are adept at altering chemical bonds and oxidizing amino acid side chains [73], thereby impacting protein functionality. The interaction between proteins and these reactive particles is confined to the protein surface, affecting areas ranging from a few to tens of nanometers. Consequently, the degree of protein modification depends on the surface area contact between the protein and reactive particles [74]. By applying plasma treatment to protein and adjusting the particle size via sieving, the specific surface area of the protein can be substantially increased, enhancing the effectiveness of the modification process [75]. A study by Eazhumalai et al. [76] highlighted the impact of oxidizing substances, such as -OH and O_3_, generated by pin-to-plate CP, and its promotion of the formation of carbonyl groups within the molecular structure of oat protein. Similarly, the effect of atmospheric cold plasma treatment, applied at levels of 60–70 kV for 5–10 min, on the structural and functional properties of both strong and weak wheat flours was examined [77]. This analysis revealed that the enhanced viscoelasticity observed in plasma-treated dough was attributed to several factors, including reactive oxygen-induced oxidation, the formation of disulfide bonds, and the modification of gluten’s secondary structure.

### 3.3. Ultraviolet Radiation

**Figure 4:**
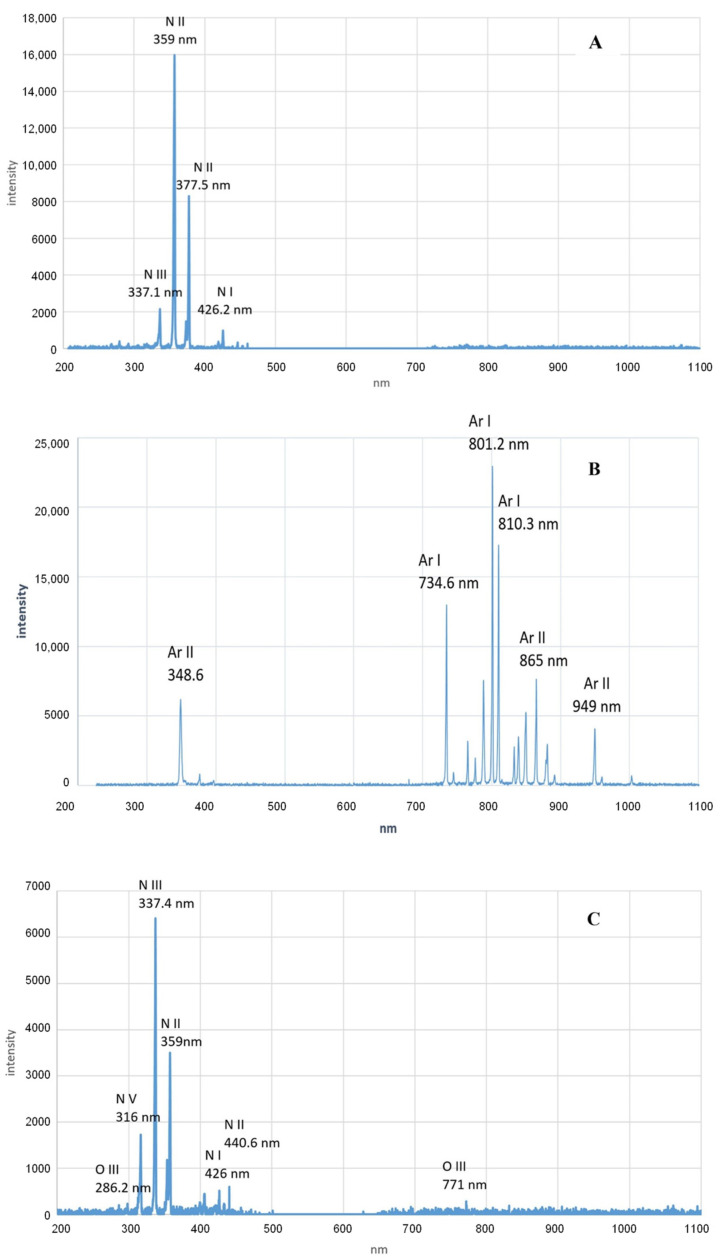
Emission spectra of dielectric barrier discharge plasma in nitrogen (**A**); argon (**B**); and ambient air (**C**). Reprinted with permission from ref. [83]. 2021 Elsevier.

Plasma formation is characterized by the emission of radiation, notably including ultraviolet (UV) and visible light [78]. Research has demonstrated that the emission spectrum of DBD-plasma in nitrogen and air predominantly falls within the UV range, spanning from 280 nm to 430 nm. Conversely, argon DBD-plasma exhibits its strongest radiation emissions in the infrared spectrum, ranging from 730 nm to 1 µm, as illustrated in Figure 4. UV photons possess a photolytic capability that can sever intermolecular bonds within proteins, thus disrupting amino acid structures and potentially leading to protein inactivation [79]. Notably, UV radiation is selectively absorbed by aromatic amino acids—Trp, Tyr, and Phe—prompting structural and chemical alterations within the proteins [80]. Kumar et al. [81] observed that UV light exposure results in modifications to both the main chain and the conformation of wheat flour proteins, influencing their functional characteristics. However, the efficiency of UV radiation in modifying proteins is inherently limited, as it is readily absorbed by air and water under standard conditions, reducing its potential for protein solution modification [82]. The production of UV radiation through CP necessitates excitation under high-pressure conditions. Low-voltage plasma treatments yield minimal UV radiation, largely absorbed by the surrounding medium, and thus have a negligible effect on proteins. Conversely, higher-voltage treatments increase UV radiation output, though this can detrimentally impact protein functionality. Therefore, the application of UV radiation for protein modification through plasma treatment is occasionally constrained.

### 3.4. Impact of pH Change

The alteration of protein pH is a notable outcome of CP treatment. In a study conducted by Mahdavian Mehr et al. [84], it was observed that the pH of grass pea protein dispersions decreased with increasing cold plasma treatment time and applied voltage. Specifically, after 600 s of treatment at 9.4 kVpp, the pH decreased from 6.60 to 5.20. Similarly, Buβler et al. [85] reported a decrease in pH from 7.0 to 4.0 when treating pea protein dispersions with CP at 8.8 kVpp for 10 min. The researchers attributed this pH change during plasma treatment to several factors: (a) the presence of reactive species in plasma, including ROS, RNS, and free radicals, directly or indirectly facilitating protein oxidation and generating acidic byproducts [86]; (b) nitrogen oxides, produced by the reaction of air under electric fields, combining with water forming nitrous (HNO_2_) and nitric acid (HNO_3_), contributing to acidity [87]; (c) bombardment by high-energy electrons or ions producing acidic entities such as hydronium ions (H_3_O^+^), further altering sample pH [88]. These physicochemical reactions induce changes in the treated sample’s pH, influencing protein behavior. Proteins, as ampholytes, exhibit minimal solubility at their isoelectric point (pI), predisposing them to denaturation precipitation [89]. A deviation from the pI by more than 0.5 pH units enhances solubility, subjecting proteins to varying degrees of denaturation under different pH conditions. In the context of plasma treatment, the pH effect is predominantly observed in liquid samples, where water ionization and interaction with CO_2_ acidify the solution, thereby modifying the proteins. In contrast, samples with low moisture content exhibit negligible pH change, limiting the impact of pH on protein modification [90].

## 4. CP Effect on Proteins and Structure

The conformational hierarchy of proteins encompasses primary, secondary, and tertiary structures, each crucial for the protein’s functional attributes. These functions can be broadly classified into interactions with water (e.g., solubility, water-holding capacity), interactions between proteins (e.g., gelation, precipitation), and surface properties (e.g., emulsification, foaming) [91]. Given that a protein’s primary structure underpins its higher-order configurations and, by extension, its functionalities, a thorough examination of protein structures is imperative for exploring functional modifications to broaden protein applications.

### 4.1. Surface Microstructure

**Figure 5:**
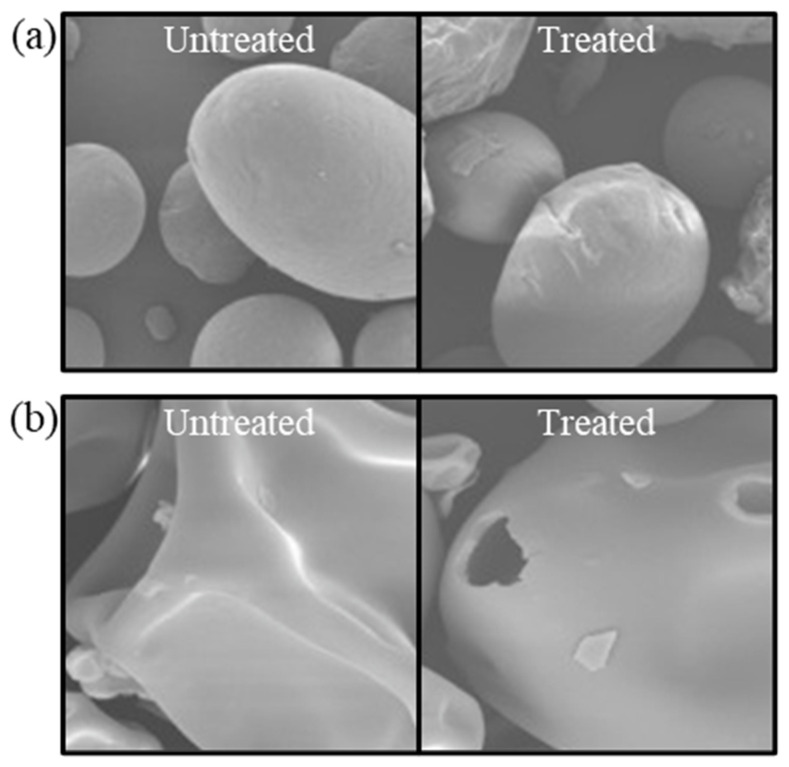
SEM images of the surface topography of untreated and plasma-treated zein (**a**) and soy protein isolate (**b**). Reprinted with permission from ref. [93]. 2017 Elsevier, ref. [94]. 2022 Elsevier.

CP treatment emerges as a potent technique for the surface modification of proteins, capable of significantly transforming their morphological characteristics. This process involves the bombardment of protein surfaces by high-energy particles under an electric field, inducing physical changes that modify the proteins’ appearance and chemical structure through oxidation reactions with amino acid residues [92]. Scanning electron microscopy (SEM) is instrumental in visualizing these alterations in protein surface microstructure post-CP treatment. For instance, Dong et al. [93] observed that corn protein isolates exhibited a rougher surface texture following atmospheric CP treatment at incremental voltages of 50 to 125 V, contrasting with the smoothness of untreated samples (Figure 5a). This textural transformation is attributed to the enhanced particle collisions at elevated voltages, which introduce irregular distortions and surface cavities. However, functionality may decline at higher voltages. Similarly, Sharafodin et al. [94] reported on the physicochemical property modifications of soy protein isolate post-DBD CP treatment (Figure 5b). The treatment intensity, measured by voltage and duration, directly influenced surface cavity formation, reflecting the dynamic interplay between high-energy electrons, ions, and plasma-generated active particles with the protein surface [95]. Nevertheless, greater surface modification did not invariably equate to improved functional properties, with optimal treatment conditions being paramount.

This physical modification through surface etching underscores the necessity for precise control over treatment conditions and protein quantity to attain desired modification outcomes. Furthermore, the relationship between CP treatment intensity and protein functionality is not linear and varies across different proteins. Therefore, optimizing plasma treatment conditions is crucial for effective protein modification, warranting comprehensive exploration to tailor treatment protocols for specific proteins.

### 4.2. Structure of Proteins

**Figure 6:**
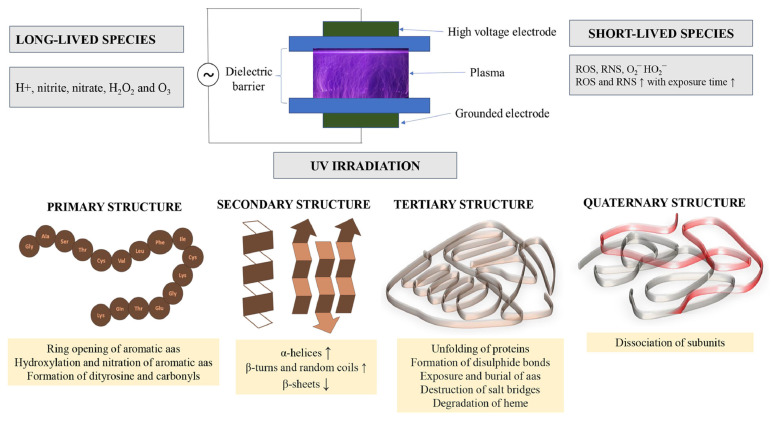
The impact of nonthermal cold plasma on the primary, secondary and tertiary structure of proteins. Reprinted with permission from ref. [101]. 2022 Elsevier.

The secondary structure of proteins encompasses the spatial arrangement of polypeptide chains into specific motifs—α-helices, β-sheets, β-turns, and random coils—primarily stabilized by hydrogen bonding. This structural level is crucial, as it underpins the formation of the tertiary structure of proteins [96]. Fourier transform infrared spectroscopy (FTIR), a pivotal bioanalytical technique [97], facilitates the investigation of protein secondary structure alterations by analyzing changes in chemical bonding, particularly within the amide I band (1600–1700 cm^−1^). This band is central to understanding protein conformation due to its sensitivity and prominence in the protein backbone [98], reflecting different structural components α-helix (1650–1660 cm^−1^), β-sheet (1618–1640 and 1670–1690 cm^−1^), β-turn (1660–1670 and 1690–1700 cm^−1^), and random coil (1645 cm^−1^) [99]. Recent research has indicated that the application of atmospheric cold plasma (ACP) can induce notable modifications in these secondary structures of proteins, as clearly depicted in Figure 6 [100]. For instance, peanut protein isolate exhibited an increase in β-turn content and a decrease in β-sheet content after 3 min of ACP treatment. Prolonged exposure led to a rise in β-sheets and random coils, whereas α-helices and β-turns diminished. This transformation suggests a shift from a tightly folded conformation to a more unfolded, expansive structure, paralleling findings from Misra et al. [77] on wheat flour treated with ACP. Such structural evolution is attributed to the etching effect of high-speed particles and the oxidative impact of active species on protein molecules, disrupting and refolding chemical bonds, thereby altering the secondary structure and consequentially, their functional attributes.

These structural adjustments have significant implications for protein functionality. Specifically, the relationship between structural elements like α-helices and β-sheets with hydrophobicity influences solubility and emulsification capacity: a decrease in α-helix content and an increase in β-sheet content typically lead to enhanced hydrophobicity, reducing solubility but improving emulsification [102]. Moreover, Xiong et al. [103] highlighted that elevated β-sheet and random coil levels could potentially improve solubility and water-holding. The unfolding of protein structures through etching and the oxidation of amino acid groups by active particles during CP treatment prompts these changes in secondary structures, directly affecting properties essential for solubility, emulsification and foam formation [104].

### 4.3. Free Sulfhydryl Groups

Sulfhydryl groups (-SH) and disulfide bonds (S-S) play pivotal roles in the functional attributes of protein-based foods, with alterations in these groups reflecting protein structure transformations [105]. The disulfide bond, a key stabilizer of protein tertiary structure, originates from the oxidation of sulfhydryl groups. Notably, cysteine within protein molecules’ amino acid side chains, characterized by its hydrophobic and aromatic groups, exhibits a high susceptibility to oxidation by active particles [106,107]. Furthermore, covalent bonds, including S-S and C-C, within proteins are particularly vulnerable to cleavage during plasma treatment due to their low cleavage energy [108]. Studies illustrate the dynamic nature of sulfhydryl group modifications under cold plasma treatment. For instance, Segat et al. [109] observed a significant decrease in free sulfhydryl groups in whey protein isolate solutions following ACP treatment, halving the -SH content after 10 min. This reduction signifies the effectiveness of ACP in diminishing cysteine sulfhydryl groups within protein structures. Similarly, Ji et al. [100] reported a 50% reduction in total sulfhydryl content in peanut protein isolate after 3 min of treatment, attributing this to the conversion of sulfhydryl groups to disulfide bonds. Further treatment led to an increase in total sulfhydryl content, as the bombardment by high-energy particles initiated the breaking and reforming of disulfide bonds into sulfhydryl groups.

These observations underscore that the evolution of sulfhydryl group content during CP treatment is complex and dependent on specific treatment conditions. Notably, the content of free sulfhydryl groups does not increase indefinitely with prolonged treatment; rather, an optimal level of plasma treatment promotes the oxidation of sulfhydryl groups into disulfide bonds, enhancing molecular stability [100,106]. Conversely, excessive treatment might reverse this process, breaking the newly formed disulfide bonds. Given the intricate effects of CP on protein structures, identifying appropriate treatment parameters for different proteins is crucial. Such optimization is essential for maximizing the modification effects on protein properties, highlighting the need for a nuanced understanding of plasma’s interactions with protein molecules.

### 4.4. Molecular Weight Distribution

Proteins are composed of multiple subunits interconnected by disulfide bonds and undergo structural and molecular weight changes upon the disruption of these bonds. Understanding these transformations is vital for elucidating protein structural modifications, for which sodium dodecyl sulfate-polyacrylamide gel electrophoresis (SDS-PAGE) is a key analytical technique [110]. SDS-PAGE, by neutralizing charge and shape variances among proteins through SDS treatment, allows for the determination of molecular weight based solely on electrophoretic mobility. Mehr et al. [111] observed notable molecular weight variations in grass pea seed protein isolate (GPPI) nanoparticles after atmospheric CP treatment for 300 and 600 s. Under non-reducing conditions, SDS-PAGE revealed not only alterations in molecular weight but also the emergence of additional subunit bands, indicative of fragmentation due to the etching effects of plasma treatment. This fragmentation was evidenced by the appearance of new bands, suggesting a breakdown of original protein aggregates into smaller fragments.

Seat et al. [106] highlighted that changes in protein molecular weight typically arise from modifications in disulfide bonds or from the oxidation of thiol groups during plasma treatment. Since proteins are primarily structured through peptide chains linked by disulfide bonds, their disruption can lead to an increase in smaller molecular subunits, while new bond formations result in larger aggregates [112]. However, these modifications do not affect the primary structure, indicating that the primary amino acid sequence remains intact. The dissociation of protein aggregates into smaller subunits, though altering molecular weight, preserves the primary structural integrity of the proteins. Therefore, controlled CP treatment can effectively unfold protein molecules, potentially enhancing their functionality without modifying their primary structure [113]. However, it is crucial to note that treatments employing higher voltages or prolonged durations might prompt the reformation of disulfide bonds among smaller subunits, thus creating larger aggregates. The impact of such reformation on the primary structure of proteins necessitates further investigation to fully understand the implications of plasma treatment on protein molecular weight distribution and functionality.

## 5. Application of CP on the Modification of Techno-Functional Properties

### 5.1. Solubility

Solubility is fundamental to the techno-functional properties of proteins in food processing, underpinning enhancements in emulsification and foaming [114]. It is influenced by hydrophobic (protein–protein) and ionic (water–protein) interactions [115], where solubility decreases as more hydrophobic residues become exposed on the protein surface [116]. Recent advancements reveal that cold plasma treatment, through modifications in proteins’ secondary and tertiary structures, can alter the exposure of hydrophobic amino acid side chains, thereby influencing solubility. Dong et al. [93] found that DBD-CP treatment of zein at various voltages (50, 75, 100, and 125 V) for 2 min enhanced solubility across all conditions, with peak solubility achieved at 75 kV. This improvement was linked to the creation of new hydrophilic groups on the protein surface, as evidenced by the concurrent trends in α-helix content and solubility. Conversely, Bußler et al. [85] observed a direct correlation between treatment duration and solubility in pea proteins treated with DBD cold plasma at 8.8 kV; a 191% increase in solubility was noted after 10 min of treatment. This was accompanied by a rise in the fluorescence intensity of tryptophan, indicating structural changes at the molecular level. 

However, the same researchers reported contrasting findings under identical conditions in another study [117]. Instead of increasing, protein solubility declined with extended treatment time, alongside a reduction in maximum fluorescence intensity. This suggested that the exposure of tryptophan from the protein’s hydrophobic core, leading to an increased presence of hydrophobic residues in the external solvent, resulted in reduced solubility. These studies underscore that the impact of CP on protein solubility is not straightforward and may vary based on protein type, concentration, treatment conditions, and pH (Table 1). Such variability highlights the necessity for tailored CP treatments to optimize protein functionality, emphasizing the intricate balance between structural modification and functional property enhancement.

### 5.2. Gelation

Gelation, the aggregation of protein molecules into an organized network, is a crucial process in food science, where the physical properties of gel-forming proteins are manipulated to produce three-dimensional gel networks. This process involves denaturation, unfolding, and aggregation of protein molecules, significantly influenced by various physical treatments. Qin et al. [118] demonstrated that microwave treatment considerably improves the gel strength of TG enzyme-induced soy protein/wheat gluten gels, leading to denser, more uniform microstructures. Similarly, Boreddy et al. [119] reported that radio frequency-assisted hot air (RFHA) treatment enhances protein gel properties more efficiently. Zhang et al. [120] noted that CP treatment of peanut protein enhances gel properties such as water-holding capacity and mechanical strength, due to an increase in surface hydrophobicity and free sulfhydryl groups.

**Figure 7:**
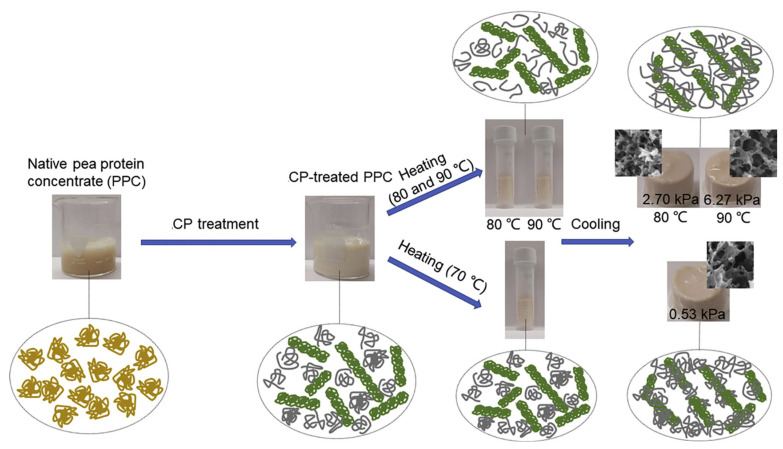
Schematic illustration of the gelling mechanism of CP enabled pea protein gelation at reduced temperatures. Reprinted with permission from ref. [120]. 2021 Elsevier.

The emerging interest in pea protein as a soy protein alternative has spotlighted its gelation behavior. While natural pea protein concentrate (PPC) does not gel effectively at 90 °C, ACP treatment allows gelation to begin at 70 °C. Although minimal protein unfolding occurs at 70 °C, it leads to weak gels with a compressive strength of 0.53 kPa (Figure 7). As the temperature increases to 80–90 °C, the unfolding of ACP-treated PPC improves, forming a strong, homogenous fibrillar network. This results in gels with improved mechanical properties, including a compressive strength of up to 2.70 kPa, 85% water-holding capacity, and good viscoelasticity (Figure 7). ACP treatment reduces the PPC denaturation temperature and enhances surface hydrophobicity and free sulfhydryl groups, facilitating stronger hydrophobic interactions and disulfide bonds, which contribute to the improved properties of the gels [120]. Rahman et al. [121] observed ACP treatment’s impact on mung bean protein isolate (MBPI) gels, lowering the gelation temperature and significantly increasing the storage modulus (G’) in 16% protein dispersions. The enhancement in gel properties was linked to a notable increase in hydrophobic interactions. Likewise, peanut protein isolate gels treated with ACP displayed improved properties, attributed to an enlarged surface area and augmented hydrophilic groups, with minimal impact from the ionic charge introduced during treatment [100,122]. Key to these improvements were the modifications in surface hydrophobicity and water distribution, crucial for the elevated water-holding capacity of the gels.

### 5.3. Emulsifying Properties

The ability of proteins to facilitate and stabilize the formation of oil droplets in emulsions defines their emulsifying properties. This function hinges on proteins adsorbing at the oil–water interface during homogenization, thereby reducing interfacial tension and preventing oil droplet coalescence through repulsive forces. These properties are determined by proteins’ surface charge, hydrophilic–hydrophobic balance, and conformational flexibility [123]. Unfolding of proteins exposes hydrophobic residues and enhances flexibility, thus improving their interfacial activity and adsorption capacity. Research has shown that the effect of CP treatment on protein emulsification depends on the applied voltage and duration. Mehr et al. [84] observed that grass pea protein emulsions treated with DBD-CP at 9.4 kV for 60 s exhibited low oil–water interfacial tension but larger oil droplet sizes, indicating an insufficiently strong interfacial layer. In contrast, treatments at 18.6 kV for 60 s yielded the smallest droplets, the highest protein adsorption at the interface, and a robust interfacial film, significantly enhancing emulsion stability. This stability improvement correlated with structural changes in proteins, including increased α-helix content, dimeric tyrosine, and surface hydrophobicity, suggesting protein unfolding and re-aggregation.

Furthermore, Mehr et al. [111] found that grass pea protein isolate nanoparticles (GPPINP) prepared under similar high-voltage conditions demonstrated superior adsorption at the oil–water interface, leading to more stable emulsions. In vitro cytotoxicity assays confirmed that GPPINP is non-toxic to normal human skin fibroblasts (NHDF), with cell viability remaining high across treatment groups. Ji et al. [122] reported enhanced emulsifying properties in peanut protein isolate following DBD-CP treatment, as evidenced by lower Turbiscan Stability Index (TSI) values indicating greater stability. Treatment led to decreased α-helix and free sulfhydryl content, increased β-sheet content, and reduced particle size, facilitating quicker diffusion and adsorption at the oil–water interface. Emulsion stability is multi-factorially determined by droplet size and distribution, protein concentration, oil type and volume, continuous phase viscosity, and storage conditions [124]. The findings collectively indicate that cold plasma treatment alters protein secondary structures, promoting unfolding, reducing droplet size, and improving surface hydrophobicity and protein adsorption, thereby enhancing the emulsifying efficacy of proteins [125].

### 5.4. Reducing the Allergenicity of Allergenic Proteins

Food allergies represent a significant public health challenge worldwide [126]. They are caused by abnormal immune reactions when immunoglobulins, such as IgE and IgG, bind to specific sites on allergenic proteins called epitopes [127]. The three-dimensional conformation of these proteins, and thus the structure of epitopes, can be altered by external factors, potentially reducing their ability to bind to immunoglobulins and decreasing the proteins’ allergenicity [128]. Cold plasma treatment has emerged as a promising method for modifying the antigenic structures of food allergens, thereby reducing their allergenicity. This process is facilitated by active substances in cold plasma that can induce structural changes in natural proteins. For example, Sun et al. [129] demonstrated that APPJ treatment reduced the immunoreactivity of wheat prolamin to 48.05% of the control, linking this reduction to modifications in the protein’s epitope structure, as indicated by the specific binding decrease of the R5 antibody to the prolamin epitope.

Similarly, Meinlschmidt et al. [37] observed that DBD and microwave CP treatments led to the disappearance of specific bands in SDS-PAGE patterns of β-conglycinin and glycinin, significantly reducing their immunoreactivity. This effect was confirmed by ELISA, with DBD treatment nearly eliminating immunoreactivity and remote (indirect) plasma treatment reducing it by 89%. Venkataratnam et al. [130] reported decreased binding of the peanut allergen Ara h1 to IgG and a reduction in antigenicity of peanut powders following DBD CP treatment, attributing these outcomes to the actions of ROS and RNS produced by the plasma. These species can attack amino acid side chains, cleave disulfide bonds, and destroy epitope structures, effectively lowering allergenicity. However, not all studies report consistent effects of cold plasma on allergenic proteins. Filho et al. [131] found no significant changes in the SDS-PAGE patterns or ELISA results for cashew allergenic proteins after low-pressure glow discharge plasma treatment, suggesting variability in cold plasma’s effectiveness may relate to the generation method and the types and concentrations of ROS and RNS produced. The divergent results across studies highlight the complex nature of cold plasma treatment and its impact on food allergens. These differences underscore the need for further investigation into how cold plasma conditions influence the reduction of allergenicity in proteins, pointing towards the potential of plasma technology in creating safer food products for allergy sufferers.

**Table 1:** A summary of different changes induced by cold plasma treatment on the properties of cereal and legume proteins.

Protein	Plasma Source	Treatment Conditions	Findings	References
Peanut protein	DBD	Input gas: air Voltage: 70 V Current: 1 A Duration: 1, 3, 5, 7, and 10 min	Solubility and water-holding capacity demonstrated a progressive increase with the extension of treatment time, achieving a peak value after 7 min	Ji et al. [100]
Grass pea protein	DBD	Input gas: air Voltage: 9.4 and 18.6 kV Current: 1 A Duration: 30 and 60 s	The interfacial and emulsifying properties of GPPI were improved after cold plasma treatment when compared to the untreated counterpart	Mahdavian Mehr et al. [84]
Soybean protein	DBD	Input gas: air Voltage: 40 to 60 kV Frequency: 50 to 150 Hz Duration: 1, 2, 5, and 10 min	The solubility of soybean protein isolates generally improved following treatment, with the emulsifying properties exhibiting an increase ranging from 56 to 168% compared to the control, and foaming properties enhancing from 60 to 194%.	Zhang et al. [40]
Flaxseed protein	Jet plasma	Input gas: air Voltage: 5 kV Frequency: 40 kHz Duration: 0, 5, 10, 15, 30, 60, 90, 120, and 240 s	The foaming, emulsifying, and in vitro antioxidant properties of flaxseed protein were significantly improved following a short-time treatment of 5 to 15 s. The relative protein solubility significantly declined after 15 s of treatment	Yu et al. [132]
Pea protein	DBD	Input gas: air and Ar + 20% O_2_ Frequency: 20 kHz Duration: 30 min	Independent of the reactive species utilized, the emulsifying properties of pea protein isolate exhibited enhancement relative to its untreated counterpart. Notably, the improvements were more pronounced when O_3_ and OH were employed for protein modification.	Bu et al. [133]
Little millet protein	DBD	Input gas: air Voltage: 60 kV Duration: 20 and 30 min	The oil and water absorption capacities of plasma-treated little millet flours exhibited an increasing trend with the augmentation of power and treatment duration. The enhanced solubility can be attributed to the oxidation of starch and molecular degradation induced by the active plasma species.	Jaddu et al. [134]
Wheat protein	DBD	Input gas: air Voltage: 80 kV Duration: 5, 10, 20, and 30 min	The oil holding capacity of all treated samples demonstrated a progressive increase with treatment time in comparison to the control.	Chaple et al. [135]
Parboiled rice flour protein	Radio frequency (RF)	Input gas: air Voltage: 30 W, 40 W, and 50 W Frequency: 13.56 MHz Duration: 5, 10 and 15 min	The oil absorption capacity exhibited a significant increase from the control to the plasma-treated samples. Additionally, water-binding capacity was enhanced following low-pressure plasma treatment, with a slight increase observed in water-holding capacity at higher plasma power and extended treatment durations.	Sarangapani et al. [136]
Pea protein	DBD	Input gas: air Voltage: 8.8 kV Frequency: 3.0 kHz Duration: 1, 2.5, 5, 7.5, and 10 min	The solubility of pea proteins was markedly enhanced following exposure to CP. The modifications in water and fat binding capacities induced by plasma treatment varied according to the exposure time and the specific composition of the matrix being treated.	Bußler et al. [85]
Pea protein	DBD	Input gas: air Voltage: 0−30 kV Frequency: 3500 Hz Duration: 0−10 min	Combined cold plasma and pH-shifting treatment enhanced the gelling capacity of pea protein by altering its structural properties and facilitating protein aggregation. These aggregates, noted for their high solubility, successfully formed robust gels upon heating at 70 °C.	Zhang et al. [137]
Soybean protein	Microwave generator	Input gas: O_2_, N_2_, air, He, and Ar Voltage: 650 W Duration: 25 min	Cold plasma treatment significantly improved both the tensile strength and moisture barrier capabilities of the films. These treated films also exhibited an increased ease of decomposition and played a crucial role in inhibiting lipid oxidation and preventing the softening of smoked salmon stored at 4 °C.	Oh et al. [138]
Zein	DBD	Input gas: air Voltage: 50 V, 75 V, 100 V, and 125 V Current: 1 A	The average diameter of zein particles decreased significantly, indicating a depolymerization of aggregations following cold plasma (CP) treatment. Additionally, cold plasma was found to enhance the tensile strength and surface hydrophilicity of the zein film in a manner dependent on the treatment voltage.	Dong et al. [93]
Pea protein	Plasma jet, DBD, nanosecond-pulsed plasma	APPJ: Ar and O_2_, 20 kHz for 5, 15, 30, and 45 min DBD: Ar with 20% of O_2_, 10.3 ± 1.1 W for 5, 15, 30, and 45 min Ns-pulsed plasma: 5, 15, and 30 min at 1 kHz and 10 kV	Different plasma sources and their associated reactive species induced protein denaturation, increased surface hydrophobicity, and led to the formation of soluble aggregates predominantly through disulfide linkages, along with alterations in secondary structures. These changes contributed to the enhancement of surface properties, the presence of soluble aggregates, and an increase in β-sheet content, all of which notably improved gelation and emulsification properties. DBD treatment for 30 min had an insignificant effect on the amino acid composition.	Bu et al. [139]
Mung bean protein	DBD	Input gas: air Voltage: 80 kV Duration: 10 min	The plasma-treated protein exhibited a minimum gelling concentration of 14%, compared to the control’s 16%. Additionally, plasma-treated protein dispersions demonstrated a storage modulus six times higher than that of the control.	Rahman et al. [121]
Chick pea	Glow discharge	Input gas: air Voltage: 40, 50, and 60 W Frequency: 13.56 MHz Duration: 10, 15, and 20 min	Plasma decreased K_1_ constant with increasing power and time.	Pathan et al. [140]
Oat protein	DBD	Input gas: air Voltage: 170 V and 230 V Frequency: 1 kHz Duration: 15 and 30 min	Plasma-induced reductions in pH and ζ-potential led to protein aggregation, while plasma-induced oxidation facilitated peptide cleavage and increased carbonyl (C=O) and thiol (F-SH) groups. Furthermore, cold plasma treatment enhanced protein solubility and decreased surface tension, resulting in improved foaming properties.	Eazhumalai et al. [76]
Wheat germ protein	DBD	Input gas: air Voltage: 25 kV Duration: 5, 10, 20, and 40 min	With extended processing time, the solubility of protein improved significantly. Notably, after a 5-min exposure to cold plasma, both the emulsifying and foaming capabilities were enhanced. Treatment parameters set at 25 kV for 5 min effectively enhanced the functional properties and served as a preventive measure against oxidative spoilage.	Abarghoei et al. [141]
Brown rice protein	Glow discharge	Input gas: helium Voltage: 400 W Duration: 5 min	Plasma treatment effectively degraded phytic acid and enhanced the levels of gamma-aminobutyric acid and γ-oryzanol in germinated rice.	Li et al. [142]
Wheat gliadin	Plasma jet	Input gas: helium + air Voltage: 20 kV Frequency: 1 kHz Duration: 0, 1, 3, 5, 7, and 10 min.	The application of plasma treatment significantly enhanced the levels of disulfide bonds and free thiol groups within gliadin. Concurrently, there was an observable increase in both the zeta potential and the size of gliadin molecules and their colloidal aggregates, which correlated with the lengthening of the treatment period. Among the plasma-treated samples, those subjected to 3 and 5 min of treatment demonstrated the most notable improvement in foam stability.	Sun et al. [143]
Peanut protein	DBD	Input gas: air Voltage: 35 V Current: 2 ± 0.2 A Time: 1, 2, 3, and 4 min	Extending the cold plasma treatment to 2 min resulted in a notable prolongation of emulsion stability. This duration specifically facilitated an increase in the free thiol (-SH) content of pea protein isolate (PPI) by inducing partial protein unfolding.	Ji et al. [122]
Whey protein	DBD	Input gas: air Voltage: 70 kV Duration: 1, 5, 10, 15, 30, and 60 min	Partial unfolding of proteins during the initial stages of plasma treatment imparts a more flexible structure, allowing better alignment at the air-water interface. Plasma treatments lasting 15 min induced mild oxidation in whey proteins, which enhanced their foaming and emulsifying capacities. Further, extending the treatment duration to 30 and 60 min resulted in increased foam stability.	Segat et al. [109]
Zein– chitosan	DBD	Input gas: air Voltage: 30, 40, 50, and 60 V Current: 1.5 A Duration: 2 min	Plasma treatment effectively reduced the particle size of the zein–chitosan complex. This reduction was facilitated by partial unfolding of the zein polypeptide chain and increased exposure of tyrosine residues, enhancing the interactions between zein and chitosan via plasma treatment. This modification also increased the availability of binding sites for carrying free resveratrol.	Chen et al. [144]
Grass pea protein	DBD	Input gas: air Voltage: 9.4 and 18.6 kV Duration: 300 and 600 s	With the increase in both treatment time and voltage, the surface hydrophobicity of GPPINPs (grass pea protein isolate nanoparticles) increased, whereas their solubility decreased. Furthermore, as the plasma treatment time and voltage were elevated, hydrophobic interactions within the GPPINPs intensified, while hydrogen bonding diminished.	Mahdavian Mehr et al. [111]
Soybean protein	DBD	Input gas: air Voltage: 16, 18, and 20 kV Duration: 5, 10, and 15 min	Soy protein isolate exhibited marked improvements in emulsifying properties, solubility, water-holding capacity, and foaming activity, reaching optimal levels after undergoing plasma treatment at 18 kV for 15 min. Moreover, extended exposure to DBD plasma at this voltage led to significant increases in the levels of both free and reactive sulfhydryl groups and free carbonyl groups, highlighting the efficacy of the treatment in modifying protein functionalities.	Sharafodin et al. [94]
Defatted peanut flour	ACP	Input gas: air Voltage: 32 kV Frequency: 52 kHz Duration: 15, 30, 45, and 60 min	With increases in plasma exposure, SDS-PAGE analysis indicated a reduction in the solubility of major peanut allergens. Modifications in the α-helix and β-sheet structures resulted in changes to the epitope binding capacity, consequently affecting antigenicity. Cold plasma treatment emerges as a promising alternative for reducing the allergenicity of peanuts.	Venkataratnam et al. [16]
Zein suspension	DBD	Input gas: air Voltage: 60, 70, 80, 90, and 100 V Current: 1 ± 0.2 A Duration: 70 s	The solubility of the protein was maximized by treating it at 70 V for 70 s. The DBD treatment disrupted covalent bonds and introduced hydrophilic groups onto the surface of the zein, thereby enhancing its contact area with water molecules. This modification facilitated a more uniform dispersion of the protein in aqueous environments.	Li et al. [145]

## 6. Conclusions and Future Trends

CP technology is emerging as an innovative, non-thermal processing approach that offers a promising avenue for modifying protein structures and enhancing their functional properties while preserving nutritional value. Characterized by its avoidance of additional chemical reagents, absence of residue production, and its energy-efficient and environmentally friendly nature, CP stands out as a compelling alternative to traditional methods of protein functionality modification. The core mechanism of action in protein modification through CP centers around the etching effect of high-speed particles on protein surfaces and their interactions with amino acid residues, crucially without altering the primary structure or amino acid composition. Furthermore, this technique uniquely prevents significant fatty acid oxidation, thus maintaining the nutritional and sensory quality of cereal and legume proteins. 

However, as the application of CP in the realm of protein modification continues to evolve, there is a pressing need for deeper exploration into the mechanisms governing its impact on protein structure and function. Current implementations of single cold plasma treatments reveal limitations in protein modification, which could be effectively addressed by combining CP with traditional modification methods, such as enzymatic treatment or glycosylation, to amplify modification outcomes. Despite its potential, the application scope of CP technology in the industry has yet to fully mature, with future research directions focusing on three critical areas: (i) the necessity for comprehensive comparative studies against traditional protein functionality modification methods to validate CP efficiency and stability as a viable alternative; (ii) the imperative for extensive safety evaluations given the complex nature of active components in food proteins treated with CP; and (iii) the need for clarity on technical conditions, including equipment, gas type, voltage, and frequency parameters, which significantly influence CP efficiency. Specifically, the choice and consumption of gas play a pivotal role in operational costs, underscoring the importance of understanding specific mechanisms of CP and establishing unified technical standards to propel its industrial application forward.

## Data Availability

No new data were created or analyzed in this study. Data sharing is not applicable to this article.
